# Bonobos Fall within the Genomic Variation of Chimpanzees

**DOI:** 10.1371/journal.pone.0021605

**Published:** 2011-06-29

**Authors:** Anne Fischer, Kay Prüfer, Jeffrey M. Good, Michel Halbwax, Victor Wiebe, Claudine André, Rebeca Atencia, Lawrence Mugisha, Susan E. Ptak, Svante Pääbo

**Affiliations:** 1 Max Plank Institute for Evolutionary Anthropology, Leipzig, Germany; 2 Lola Ya Bonobo Bonobo Sanctuary, “Petites Chutes de la Lukaya”, Kinshasa, Democratic Republic of Congo; 3 Réserve Naturelle Sanctuaire à Chimpanzés de Tchimpounga, Jane Goodall Institute, Pointe-Noire, Republic of Congo; 4 Chimpanzee Sanctuary and Wildlife Conservation Trust (CSWCT), Entebbe, Uganda; Université de Toulouse, France

## Abstract

To gain insight into the patterns of genetic variation and evolutionary relationships within and between bonobos and chimpanzees, we sequenced 150,000 base pairs of nuclear DNA divided among 15 autosomal regions as well as the complete mitochondrial genomes from 20 bonobos and 58 chimpanzees. Except for western chimpanzees, we found poor genetic separation of chimpanzees based on sample locality. In contrast, bonobos consistently cluster together but fall as a group within the variation of chimpanzees for many of the regions. Thus, while chimpanzees retain genomic variation that predates bonobo-chimpanzee speciation, extensive lineage sorting has occurred within bonobos such that much of their genome traces its ancestry back to a single common ancestor that postdates their origin as a group separate from chimpanzees.

## Introduction

**Figure 1:**
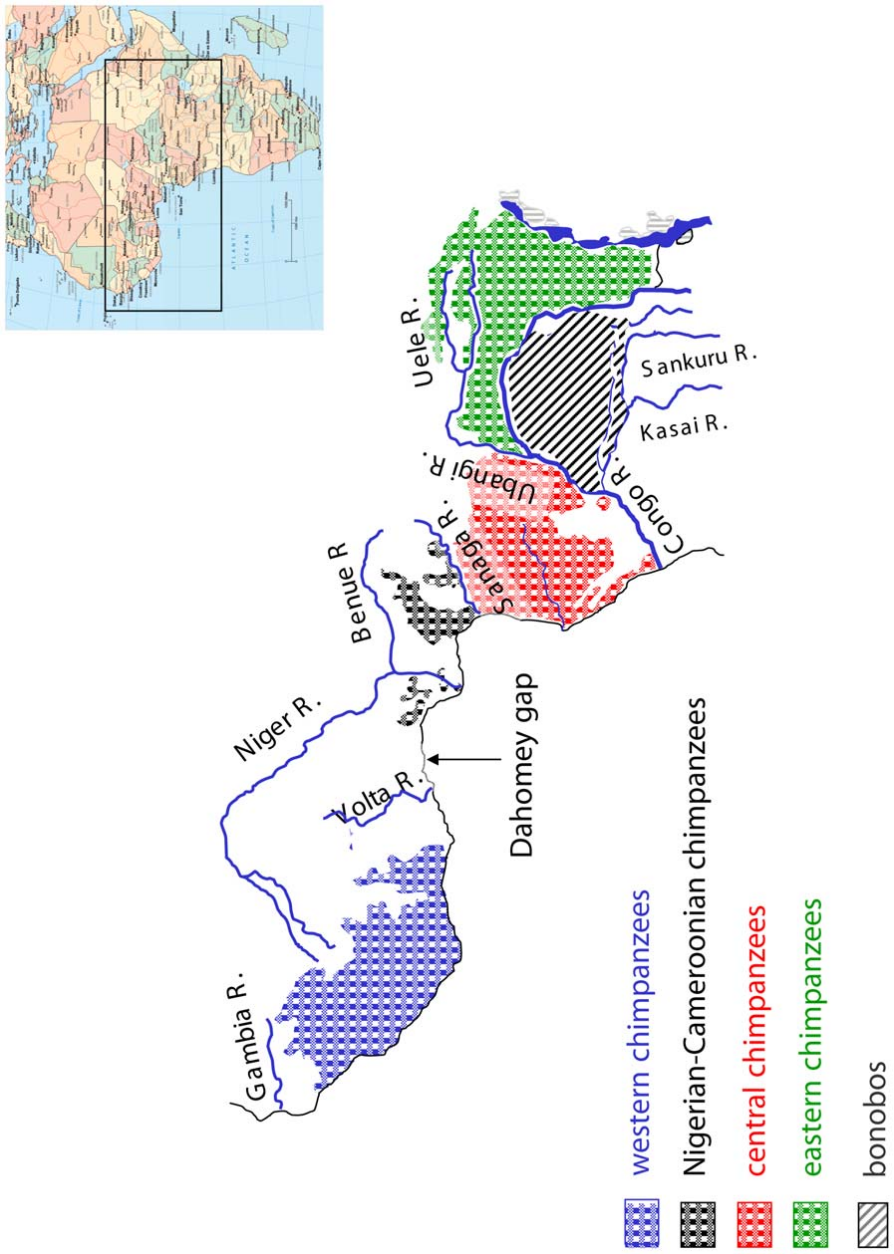
Map of population ranges.

In humans, extensive data has been collected for large numbers of individuals (e.g. [1]; HapMap project, www.hapmap.org; NIEHS SNP project, http://egp.gs.washington.edu/; the 1000 genomes project). By contrast, data on DNA sequence variation in the closest non-extinct relatives of humans, chimpanzees (*Pan troglodytes*) and bonobos (*Pan paniscus*), are limited to a handful of studies that have collected genetic data across a handful of genomic regions to discern basic population and demographic parameters [2], [3], [4], [5], [6], [7]. While these studies provide an important first step, their geographic sampling was limited, so they are likely to have captured only a fraction of the species-wide variation that may exist within natural populations. In particular, variation in bonobos and some populations of chimpanzees has been examined in only a few studies of mostly captive-born individuals [6], [8]. In chimpanzees, four “subspecies” are commonly recognized which correspond to the geographic ranges where these groups are found (Figure 1): western chimpanzees (*Pan troglodytes verus*); Nigerian-Cameroonian chimpanzees (*P.t. ellioti* or formerly *P.t. vellerosus);* central chimpanzees (*P.t. troglodytes)*; and eastern chimpanzees (*P.t. schweinfurthii*). Little or no morphological and behavioral differences distinguish the four groups from each other [9], [10], [11]. In contrast, bonobos (*Pan paniscus*) have no recognized subspecies but clearly differ from chimpanzees in morphology and behavior [11], [12], [13], [14]. Genetic variation in bonobos has been examined in only a few, usually captive-born individuals [2], [3], [4], [5], [6], [15], [16]. We have collected DNA from 20 wild-born bonobos and 44 wild-born and 14 captive-born chimpanzees representing all four chimpanzee groups (see Materials and Methods) and sequenced 15 non-coding autosomal regions each encompassing about 10,000 basepairs (bp), as well as the complete mitochondrial (mt) DNAs from these individuals.

## Results and Discussion

### Genetic diversity

To characterize the patterns of genetic diversity within groups, we computed various summary statistics (Table 1). As previously shown [6], [8], [17], [18], [19], nucleotide diversity and hence effective population sizes are highest in central and eastern chimpanzees and lowest in western chimpanzees, while bonobos have diversity levels similar to western chimpanzees, in agreement with previous studies of zoo [2], [6] and wild-caught individuals [20]. Diversity levels in Nigerian-Cameroonian chimpanzees are intermediate to those of central and eastern chimpanzees on the one hand and western chimpanzees and bonobos on the other.

**Table 1:** Summary statistics for 15 autosomal regions and mitochondrial DNA.

		# of chromosomes	length $	S $$	π (%)	θw (%)	*N_e_*	Tajima's D	Fu and Li's D*	Fit to standard neutral
autosomal regions										
Bonobos		40	144055	611	0.09	0.10	11100	−0.37	0.01	0.0396
Chimpanzees	central	40	140718	1908	0.24	0.32	35400	−0.90	−1.04	0.000*
	eastern	40	138916	1239	0.21	0.21	23400	−0.02	0.42	0.283
	western	28	142044	435	0.08	0.08	8900	−0.15	0.11	0.936
	Nigerian-Cameroonian	8	142544	746	0.20	0.20	22400	−0.15	−0.12	
mtDNA										
Bonobos		20	16552	279	0.44	0.48	−	−0.30	−0.20	
Chimpanzees	central	20	16511	453	0.52	0.78	−	−1.37	−1.90	
	eastern	20	16463	212	0.21	0.36	−	−1.76	−2.42	
	western	14	16546	287	0.69	0.56	−	1.05	0.84	
	Nigerian-Cameroonian	4	16555	83	0.27	0.27	−	−0.22	−0.22	

$ number of aligned bases excluding alignment gaps.

$$ number of SNPs.

*significant values p<0.05

The p-value for the fit to the standard neutral model was assessed using the method of [21]. This p-value can be interpreted as the proportion of simulated data sets that gave a composite test statistic more extreme than observed.

We assessed how well a null model of constant population size and random mating fit each population [21]. Only the central chimpanzee samples deviate from this null model (p<1/10000) due to an excess of rare alleles (Table 1 and Table S1) that suggests population growth as has been previously reported [3], [4], [22].

### Genetic relationship between populations

#### Chimpanzee populations

**Figure 2:**
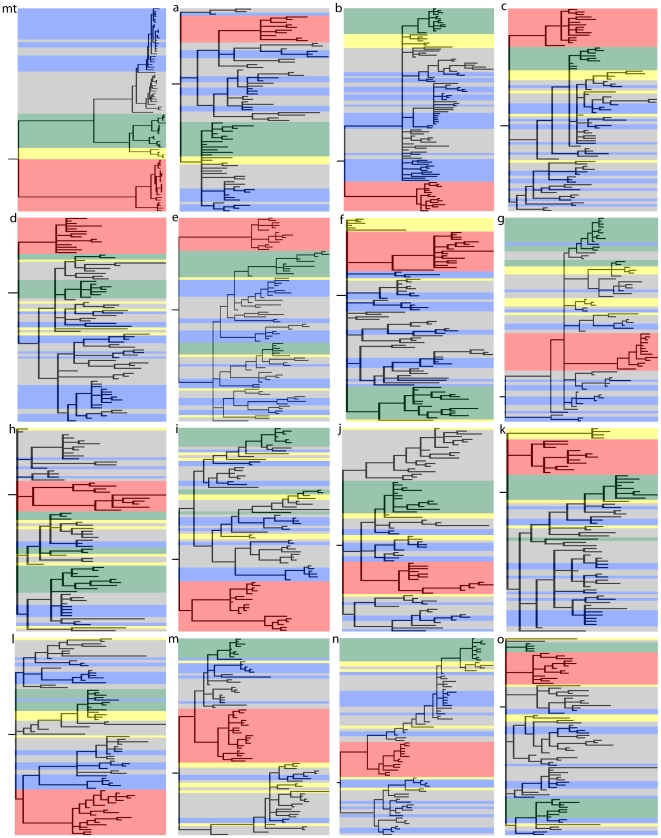
50% majority consensus tree for mtDNA (mt) and each of the fifteen nuclear regions (a to o). Colors: red for bonobos, green, grey, blue, and yellow for western, central, eastern and vellerosus chimpanzees, respectively.

Figure 2 shows the phylogeny estimated for each genomic region. While central, eastern and Nigerian-Cameroonian chimpanzees are highly interspersed in these phylogenies and never form monophyletic groups, western chimpanzees are monophyletic in 6 of the 16 trees (Figure 2; Table 2). As expected from its lower effective population size and as has previously been reported based on smaller numbers of individuals and regions [20], mtDNA shows more clustering than the autosomal DNA sequences. We note that four individuals designated as central chimpanzees fall within the eastern chimpanzee cluster for mtDNA. Three of these individuals were confiscated in the Democratic Republic of Congo, where both central and eastern chimpanzees can be found. Below, we report the results with all individuals included but have checked that the results are not significantly changed when these four individuals are excluded from the analyses wherever necessary.

**Table 2:** For each region, the posterior probability for a tree that supports (1) reciprocal monophyly of chimpanzees and bonobos (2) and monophyletic grouping of bonobos (3) chimpanzees as a whole (3–6) each population of chimpanzee separately.

Region	Reciprocal monophyly	Monophyly of					
		Bonobos	Chimpanzees	Western chimpanzees	Nigerian-Cameroonian chimpanzees	Eastern chimpanzees	Central chimpanzees
mtDNA	1	1	1	0.999	1	0	0
a	0.003	1	0.003	0.002	0	0	0
b	1	1	1	1	0	0	0
c	0.494	1	0.494	0.077	0	0	0
d	0.955	1	0.955	0	0	0	0
e	1	1	1	0	0	0	0
f	0.223	1	0.223	0.956	0	0	0
g	0	1	0	0	0	0	0
h	0.076	0.913	0.081	0	0	0	0
i	0.946	0.957	0.989	0	0	0	0
J	0.005	1	0.005	0.607	0	0	0
k	0.375	0.998	0.377	0	0	0	0
l	0.077	1	0.077	0.002	0	0	0
m	0.002	1	0.002	1	0	0	0
n	0	0.999	0	1	0	0	0
o	0.163	1	0.163	0	0	0	0

To compute this posterior probability, we counted in how many trees from the posterior distribution was a given population monophyletic, and in how many trees were both bonobos and chimpanzees monophyletic. For the text, we arbitrarily defined a tree as showing support for monophyly if the posterior probability >95% and as showing support for paraphyly if the posterior probability <5%.

**Figure 3:**
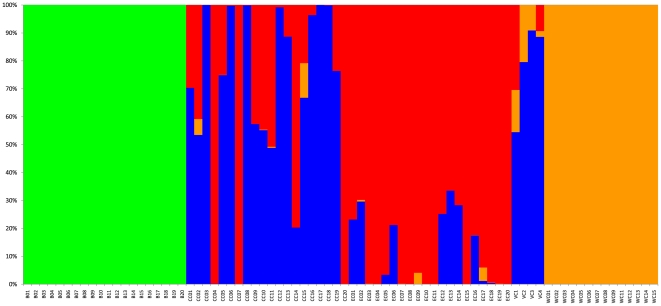
Plot of inferred ancestry for each individual assuming four populations, as assessed by the program Structure. The inference was done blind to actual population labels. Individuals: 1 to 20 are bonobos, 21 to 40 central chimpanzees (with individuals 24, 27 and 40 being the ones confiscated in DRC), 31 to 60 eastern chimpanzees, 61 to 64 Nigerian-Cameroonian, 65 to 78 western chimpanzees. Colors: green for bonobos; orange, blue and red for western, central, and eastern chimpanzees, respectively.

**Figure 4:**
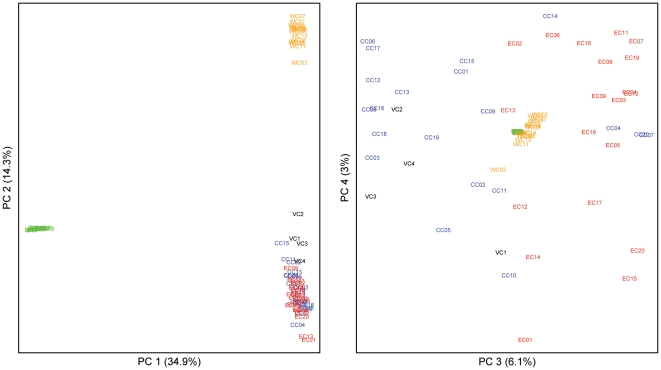
Principle Component Analysis (PCA) including all individuals. PC1 separates bonobos from chimpanzees, PC2 western chimpanzees from other chimpanzees, PC3 and PC4 are gradients of genetic variation. Colors: green for bonobos; orange, blue, red and black for western, central, eastern and Nigerian-Cameroonian chimpanzees, respectively. The significance of PC3 does not depend on the inclusion of the three chimpanzees confiscated in Democratic Republic of Congo (results not shown).

When we combine all autosomal SNPs, the level of genetic differentiation between pairs of chimpanzee populations (as assessed by F_st_) was highest for comparisons involving western chimpanzees (Table 3). When the program *Structure* was applied to the whole dataset, the highest likelihood was obtained for the model with four populations (Figure 3), the four populations being bonobos, western, central, and eastern chimpanzees. While the picture is clear for western chimpanzees, it is more complex for central and eastern chimpanzees: six eastern chimpanzees are inferred to have more than 20% ancestry from central chimpanzees and among the 16 central chimpanzees that according to the mtDNA are of central African origin, seven have more than 20% ancestry from eastern chimpanzees. In a principal component analysis (PCA) using all individuals (Figure 4), the first PC separates bonobos from chimpanzees while the second PC, that explains 14.3% of the variation, separates western chimpanzees from the other chimpanzees (p<10^−8^) (Figure 4). The third PC, explaining 6.1% of the variation, tend to separate eastern and central chimpanzees. Note that the four central chimpanzees falling with eastern chimpanzees on the right of the graph are the same individuals that group with eastern chimpanzees in the mtDNA phylogeny.

**Table 3:** Pairwise F_st_ values.

	bonobos	eastern chimpanzees	central chimpanzees	western chimpanzees
bonobos	-	-	-	-
eastern chimpanzees	0.56	-	-	-
central chimpanzees	0.54	0.07	-	-
western chimpanzees	0.74	0.42	0.38	-
Nigerian-Cameroonian chimpanzees	0.59	0.21	0.16	0.37

Of particular interest is that none of the autosomal phylogenies support the monophyly of the Nigerian-Cameroonian chimpanzees (Figure 2; Table 2). In fact, Nigerian-Cameroonian individuals group with individuals from all other three populations (Figure 2). The F_st_ based on the autosomal sequences show that Nigerian-Cameroonian chimpanzees are more differentiated from western chimpanzees (F_st_  = 0.37) than from central and eastern chimpanzees (F_st_  = 0.16 and 0.21, respectively) (Table 3) and the *Structure* analysis fails to suggest that the Nigerian-Cameroonian chimpanzees are a separate population (Figure 3), and instead suggests that they have more than 50% ancestry shared with central chimpanzees. Likewise, in the PCA, the four Nigerian-Cameroonian individuals fall within central and eastern chimpanzee variation, while being separated from western chimpanzees (Figure 4).

An earlier study of one wild-caught Nigerian-Cameroonian individual showed it to be related to both western and central chimpanzees based on nuclear microsatellites [2]. However, in a recent study [7] of 94 chimpanzees, including 32 designated as Nigerian-Cameroonian, the weight of evidence is supporting three major groups, which are western chimpanzees, Nigerian-Cameroonian chimpanzees and central/eastern chimpanzees. One possible reason for this apparent discrepancy may be the limited number of Nigerian-Cameroonian individuals in our study. However the nuclear DNA sequences studied here are expected to reflect historic events over a much greater time depth than microsatellites. Thus, our data suggest that any genetic differentiation of Nigerian-Cameroonian populations is likely to have occurred relatively recently in the evolution of chimpanzees. Regardless, it is important to note that both nuclear data sets fail to support the close relationship of Nigerian-Cameroonian chimpanzees and western chimpanzees observed for mitochondrial DNA (Figure 2) [23], [24], [25].

### Chimpanzees and Bonobos

When all SNPs in the nuclear data are combined, more than 50% of the variation is between bonobos and chimpanzees (0.54≤F_st_≤0.74; Table 3) and the *Structure* (Figure 3) as well as the PCA analyses (Figure 4) support a separation between bonobos and common chimpanzees as does a concatenated phylogenetic analysis of all of autosomal region (Figure S1). However, the individual autosomal phylogenies reveal contrasting patterns between bonobos and chimpanzees. Bonobos are strongly monophyletic (*i.e*., all bonobos are more closely related to each other than to any chimpanzee) for all but one genomic region (Figure 2; Table 2). The one possible exception (Figure 2 h) also shows a high posterior probability for the bonobo clade (P = 0.913). Thus, much of the bonobo genome coalesces to a common ancestor since the population split from chimpanzees. In contrast, monophyly of all chimpanzees is only observed in a small subset of the phylogenies (4 out of 16) and for 5 of the 16 regions bonobos unambiguously fall within the diversity of chimpanzees (*i.e*., some chimpanzees are more closely related to bonobos than to other chimpanzees) (Figure 2). This is also reflected in the patterns of allele sharing (Table S2) where there are more sites that unite bonobos to the exclusion of chimpanzees (than vice versa) and more sites for which bonobos fall within the diversity of chimpanzees (than vice versa).

Phylogenetic lineage sorting subsequent to the separation of two species necessarily proceeds through three distinct phases: complete lack of sorting (polyphyly), sorting within one species (paraphyly), and complete sorting (reciprocal monophyly) [26], [27], [28], [29]. Incomplete lineage sorting has been seen for many other closely related organisms (*e.g.* [30], [31], [32], [33]). In the human genome, about 32% of regions share a more recent ancestry with gorilla than with chimpanzees or fall outside chimpanzees and gorillas [34], [35], and for ∼1% of the genome, humans may share a more recent ancestry with either chimpanzees or bonobos rather than these with each other [3]. We show that chimpanzees retain ancestral polymorphisms to a much greater degree than bonobos. This is presumably a result of a larger effective population size of the chimpanzees, as reflected in the estimates of diversity, which for central chimpanzees (the most diverse population) suggest an effective size of ∼35,000 and an average coalescent time of DNA sequences of 2.8 million years ago (*i.e.* ∼4*N*_e_ generations ago). In contrast, the effective population size of bonobos is ∼11,000 which results in an average coalescent time of 880,000 years ago.

## Materials and Methods

### Collection of sequence data

#### Ethics statement

All animal work was conducted according to relevant national, EU and international guidelines. In all cases, the animals were not subjected to any experimental procedures, and the blood samples used were left-over aliquots collected by veterinarians carrying out routine medical examination. Authorization for use of the samples was obtained from the respective Ministries of Environment as well as by the Ministère de la Recherche Scientifique (DRC) to “Les Amis des Bonobos du Congo”, the Uganda Wildlife Authority and the Uganda National Council for Science and Technology, and the Ministère de l'Enseignement Supérieur et de la Recherche Scientifique from Republic of Congo. The international transport of samples was approved (CITES numbers: Uganda E-3520/05, Kenya E-1259/05, DRC E-0908/07, Republic of Congo E-1274/07). The proposal that in part cover this research (233297, TWOPAN) was reviewed and approved by the European Commission.

### Samples

A total of 58 unrelated common chimpanzees and 20 unrelated bonobos were used for this study. Most of these apes were confiscated by various officials from individuals selling these animals for trade or who kept them as pets, and then were brought to the sanctuaries. Where an animal is confiscated is thought to be an imperfect, but probable indication of where the chimpanzee was originally living and the population identity of the chimpanzees are mostly based on this. We thus have a sample of 20 eastern chimpanzees (*Pan troglodytes schweinfurthii*) from Ngamba island sanctuary, Entebbe, Uganda, 20 central chimpanzees (*Pan troglodytes troglodytes*) from Tchimpounga rehabilitation center, Pointe-Noire, Republic of Congo, 14 western chimpanzees (*Pan troglodytes verus*) either wild-born in Sierra Leone or captive born from individuals originating from Sierra Leone, four Nigerian-Cameroonian chimpanzees (*Pan troglodytes ellioti* or formerly *P.t. vellerosus*) from Sweetwaters chimpanzee sanctuary, Kenya and 20 bonobos (*Pan paniscus*) from Lola ya bonobo sanctuary, Kinshasa, Democratic Republic of Congo. Nigerian-Cameroonian chimpanzees were confiscated in Cairo, Egypt, and originated from Nigeria. An analysis of mtDNA confirmed their designation as Nigerian-Cameroonian chimpanzees. Since the sampling scheme follows the broad population framework established based on geography and analysis of mtDNA, our analysis does also [23], [36].

The blood samples from central and eastern chimpanzees and bonobos were collected by Michel Halbwax and Anne Fischer in 2007 and 2008 during regular health checks. The lymphocytes were extracted from blood samples using a Ficoll gradient and frozen.

### Genomic regions chosen

For each individual, we targeted the complete mitochondrial genome and 15 regions of approximately 10 kilobases (kb). The 15 nuclear regions are all non-coding, distant from known genes (at least 72 kb) and were chosen to have average recombination rate and GC content in the human genome. Based on these criteria, we used eleven of the 15 nuclear regions that overlapped with locus-pairs previously sequenced in humans [21]. Four additional regions were picked at random in the genome, based on the same criteria. The chromosomal location and coordinates are given in Table S3. Based on human recombination estimates, the average recombination rate for all regions was 1.63 cM/Mb, slightly higher than the human genome-wide average of 1.19 cM/Mb. The average GC content for these regions was 37.94%, a bit lower compared to the genome average in humans of 42%. Human was used as an outgroup (genome sequence built hg18).

### DNA extraction, amplification and sequencing

DNA was extracted from 50 ml cell cultures (5–50×10^6^ cells) obtained from lymphocytes transformed with Epstein-Barr virus using the Gentra-puregene kit from QIAGEN and following manufacturer's instructions. The DNA was aliquoted to a concentration of 100 ng/µl for further use.

For the mitochondrial genome, two sets of primers previously designed to amplify mtDNA from a large number of primates were used [37]. Each of the 15 regions was amplified in a single polymerase chain reaction (PCR) using primers that were designed from the reference human and chimpanzee genome sequences. All loci were amplified in 50 µl reactions and PCR was run with an annealing temperature of 64°C according to the manufacturer's instructions. PCR products were cleaned with PEG purification, washed twice with 70% ethanol, and eluted in TE.

All sequencing was performed using the 454 FLX sequencing platform. We used a parallel tagged sequencing protocol [37] to enable multiplexing of regions and individuals.

### Assembly

We used an iterative mapping assembler, (MIA, R. E. Green, http://sourceforge.net/projects/mia-assembler/) to assemble all reads. The first round of MIA performs a mapping assembly based on a reference genome, which for the 15 autosomal regions was the human genome sequence and for the mitochondrial genome the published sequences for bonobo and chimpanzee, respectively. MIA then uses the consensus-call of the previous round of alignments as reference for further rounds of alignment until the called consensus sequence is not changed in two consecutive rounds. MIA was run separately for each individual and for each region. However, initially all reads from a given individual were included during the assembly of each region. If a read was aligned to more than one region, the best alignment score was used to assign the read to only one region. All regions were then assembled again using MIA, but using only those reads that matched each region best. To be considered for the assemblies, at least 12 consecutive base-pairs of a read had to match the reference sequence. An average of six percent of the reads did not map to any reference sequence and likely represents unspecific PCR products. To minimize the effect of homopolymer over- and under-calls in 454 data, we used a gap penalty that decreased according to the function 1/L, where L is the homopolymer length. To test whether using the human reference sequence as the initial mapping sequence influences the outcome of the iterative procedure, all autosomal regions from one bonobo were assembled using both the human and chimpanzee sequences as reference. The final consensus sequences were identical except for the length of nine different homopolymers (all of length >6), six cases where simple repeat regions differed in length and lead to misaligned reads, and two cases where there was an insertion in the chimpanzee (114 and 214 base pairs, respectively) not present in the human genome. We therefore masked simple repeat regions from further analyses. Our analyses were not affected by homopolymer over- or undercalls or by insertion-deletions, as these were ignored.

### Calling of SNPs

We used perl scripts to identify potential heterozygous sites within a given individual from the MIA output file. Below, we list the filtering steps that were applied to get a set of high-quality SNPs.

1. In order to remove multiple sequences that may be generated from single molecules being amplified in an emulsion droplet with more than one bead (*i.e.* emulsion PCR duplicates) [38], we retained only the read with the highest quality score for each group of reads with identical strand and start position in the alignment.
2. Following [39], we filtered all reads whose alignment to the consensus sequence contained gaps within 5 base-pairs on each side of the potential SNP position.
3. We allowed at most one mismatch in 5 neighboring bases around the potential SNP.
4. We did not call SNPs if there exists a homopolymer of length longer or equal to 6 within a 20 base pair window around the site, since we observed high rates of sequencing error and misalignments in these regions.
5. Following [39], we also used the quality scores produced by the 454 base calling software (Version 2.0) to apply the Neighbor-Quality Score (NQS) with a cutoff of at least 15 for 5 neighboring positions on each side of the potential SNP position and 20 for the middle base on all reads.

If, after removal of reads due to filters 1 and 2, the potential SNP position was covered by less than 8 reads, or if the potential SNP position failed due to filters 3, 4 and 5 then we were unable to call a SNP in this position. If we were able to call a SNP, then we considered the potential position to be a SNP (*i.e.* to be heterozygous) if the minor allele frequency was above 0.15 and at least one read from each allele passes the NQS criteria outlined in filter 5. Otherwise the potential position was considered to not contain a SNP (*i.e.* to be homozygous).

### False positive rates in homozygous PCR products

We used data from ten 5 kb X chromosomal regions (Thalmann et al., in preparation) collected in a similar fashion to our data in two male humans to test the false positive rate of our SNP calling protocol. No SNPs were called.

### False negative rates by comparing 454 data to previously sequenced data

In order to further test how many heterozygous positions were missed or gained, we compared the newly generated 454 data with data previously generated by Sanger sequencing [6]. Seven samples from western chimpanzees and 9 regions were overlapping between this study and the one from [6], representing a total of ∼9 kb of data. This 9 kb contained 62 SNPs previously identified with Sanger sequencing. We found all of them when applying our filtering criteria. We also found one more SNP, which we did not call with Sanger sequencing.

### Effect of changing SNP calling algorithm

We varied the filtering criteria and looked at how the number of inferred SNPs changed for the above two situations. The filtering criteria chosen were the ones that gave the least number of false positives and false negatives. Using more permissive values resulted in finding SNPs when there were none (or were not present under Sanger sequencing), while more restrictive values made us lose SNPs which were present under Sanger sequencing.

Furthermore, our nucleotide diversity estimates and the excess of rare alleles in central chimpanzees (see Table 1) all confirm previous findings (e.g. [4], [6]). This suggests that we are not excessively missing rare alleles or overcalling SNPs, at least compared to Sanger sequencing.

### Homozygous regions/allelic dropout

When analyzing the data from the 20 bonobos, 85 of the 300 products (15 loci * 20 individuals  = 300 products) were homozygous across the entire 10 kb of sequence. This raised the concern of allelic dropout in our data. To exclude allelic dropout, we repeated the entire experiment for bonobos with nested primers. One region turned out to be affected by allelic dropout. For all other samples, we thus repeated all the regions that were completely homozygous in one individual with a second pair of primers. We note, however, that we did observe individuals in both bonobos and chimpanzees that seem to be truly homozygous across some 10 kb stretches of DNA.

In the case of no allelic dropout and equal amplification of both alleles in the PCR, we expected both alleles at a frequency close to 50% and the distribution of the minor allele frequency to look like half a normal distribution. A closer look at the data revealed that for some heterozygous individuals the minor allele frequency was skewed. This can be explained by unequal amplification of both alleles, which could be due to a mismatch to one allele in the 3′ end of one primer. We therefore plotted the minor allele frequency for all regions for each individual separately and also repeated each region showing a skew in the minor allele frequency with a second pair of primers. A skew was defined as the minor allele frequencies of each SNP in one region are all below 30%. Figure S2 shows the shift in minor allele frequencies before and after using a new set of primers for one population.

We note that 6 products still showed a skew in minor allele frequency after using different primers and repeating the PCR. We kept them as is in the analysis.

### Sequence analyses

The consensus sequences for each region were aligned with Muscle using default parameters [40], [41].

Sequences are available under accession numbers JF725992 - JF727238.

### Population genetic analyses

Summary statistics were calculated using DNAsp v5 [42], including nucleotide diversity (π and θ_w_), Tajima's D, Fu and Li's D*. Effective population sizes (*N*_e_) were estimated as *N*_e_ = θ_w_ /4 µ [43], where µ = (*d*/2*t*)*g* [44], *d* is a sequence divergence of 1.35% as estimated from the data, *t* the time since divergence between humans and chimpanzees (6 million years), and *g* the generation time assumed to be 20 years [4].

### Testing the fit of each population to standard neutral model

We tested the fit of each population to a standard neutral model based upon the observed allele frequency spectra using the method of [21]. Briefly, we used the program ms [45] to simulate 1,000 15 locus datasets, where for each locus we matched the total number of chromosomes and the average length (∼10,000 bp) in a given population. θ and ρ for each locus were chosen from a distribution. Values for θ followed a gamma distribution parameterized using the average and variance in the mutation rate across all 15 loci. Mutation rates were estimated based on divergence to human, assuming a generation time of 20 years (Fischer et al. 2004) and a divergence time of 6 million years. Values for ρ followed a log-normal distribution parameterized using the average human recombination rate and variance in the human recombination rates for all 15 loci [46].

The observed and simulated data were compared using the variance across loci of Tajima's D plus four additional summary statistics whose average value across all loci were computed: the number of segregating sites (S), the mean pairwise difference (π), Fu and Li's D*, and Tajima's D. For the simulated data, these summary statistics were computed using sumstats [47]. Following [21], we computed the probability of observing each summary statistic across the ∼1,000 simulated datasets and then calculated the sum, C, of the natural log of the p-values for each of the summary statistics.

We evaluated the fit of a given demographic model by calculating the probability of observing C in the simulated data. Note that our approach differs from [21] in that we do not include the population recombination rate, ρ, in our list of summary statistics. Independent estimates of local recombination rates, which are often not well conserved between humans and chimpanzees [48], [49], are not yet available in chimpanzees and bonobos and our data generally provided a poor fit across populations to recombination estimates derived from human populations (data not shown).

### Population structure

To explore genetic structure among populations, we used two approaches. The Structure software [50] was run using the admixture model, so that individuals were allowed to have ancestry from multiple populations. Three independent runs were performed with a model of correlated allele frequencies, a “burn-in” of 100,000 Markov Chain Monte Carlo (MCMC) iterations, and 1,000,000 additional MCMC iterations. The number of populations assumed, K, varied from 2 to 7. We averaged the results of the three independent runs for each K value to determine the most likely model, *i.e*. the one with the highest likelihood.

In addition, the Eigensoft software package [51] was used to perform a principal component analysis (PCA). For pairs of SNPs in high linkage disequilibrium (r^2^>0.5), one position was randomly excluded. Likewise, we removed all positions with a minor allele frequency lower than 5%. The statistical significance of any given principle component (PC) is obtained by a bonferroni-corrected Tracy Widom test [51]. A significant PC was considered indicative of significant population structure, which can be in the form of clusters or gradients along an axis of genetic variation.

### Population divergence times

We attempted to use MIMAR [17], a Markov Chain Monte Carlo approach which allows for some recombination to estimate divergence times and migration rates between closely-related populations, on each pair of populations. However, we found that the reasonably high recombination rates within our 10 kb regions proved too computationally demanding and we failed to reach convergence of the Markov chains even after four months.

### Phylogenetic analysis

We reconstructed the phylogeny of each region, using Bayesian inference as implemented in MrBayes v3 [52]. Each region was collapsed to unique reconstructed haplotypes and a best-fit model of sequence evolution was selected using decision theory with the program DT-ModSel [53] and PAUP* v4.0d105 [54]. However, only a few models are available in MrBayes. Thus, we chose the closest best fit model that is actually implemented in MrBayes. For mtDNA, the 14 nuclear regions and all regions concatenated the closest best-fit model was the Hasegawa-Kishino-Yano substitution matrix [55] with invariant sites and a gamma distributed correction for rate heterogeneity (HKY+I+G) [55]. For one region the general-time reversible (GTR+I+G) model was the best model. (No qualitative differences were seen if we used the (HKY+I+G) model for all regions). For each region, we ran four independent runs, each for ten million generations and sampled every 1,000 generations. For each run, we used one cold and three heated Markov Chains. We excluded the first 10% of each run, resulting in a posterior distribution of 36.000 distinct tree topologies. We used the program Tracer v1.4.1 (http://tree.bio.ed.ac.uk/software/tracer/) to verify that convergence was reached by the chosen burn-in. A single human sequence was used as an outgroup for all phylogenetic analyses. We calculated the posterior probability of monophyly by determining the proportion of phylogenies for a given locus that were consistent with monophyly for each population group and/or species. To do this, we used PAUP* to constrain the posterior distribution of phylogenies from the four independent runs of MrBayes (minus the 10% burn-in for each run) to conform to each of the following hypotheses: monophyly of bonobos, western chimpanzees, eastern chimpanzees, central chimpanzees, Nigerian-Cameroonian chimpanzees, all chimpanzees, and reciprocal monophyly between chimpanzees and bonobos. The proportion of phylogenies retained under each constrained model was taken as the posterior support for each hypothesis. We arbitrarily defined a tree as showing support for monophyly if the posterior probability >95% and as showing support for paraphyly if the posterior probability <5%.

We also estimated a single phylogeny for a concatenated alignment of the 15 nuclear regions. We had difficulty reaching convergence of the Markov Chains in our initial analyses using a Bayesian framework. Therefore, we estimated a single phylogeny with Maximum Likelihood (ML) using the program RAxML (v. 7.2.8; [56]). We used the fast bootstrapping algorithm under the GTR+G model of sequence evolution. Two thousand bootstrap replicates were performed with simultaneous optimization of the ML topology.

## Supporting Information

Figure S1 **Maximum Likelihood consensus tree based on the concatenated sequences of all 15 regions, with bootstrap values for 2000 replicates.**
(TIF)

Figure S2 **Minor allele frequency distribution for eastern chimpanzees before (a) and after (b) reamplification with a new set of primers.**
(TIF)

Table S1 **Simulated values of various summary statistics under the standard neutral model matched for S and the number of chromosomes.** Also listed is the observed value for each summary statistic.
(DOC)

Table S2 **For each region, the number of sites for which (1) chimpanzees are polymorphic and bonobos are fixed for the derived state; (2) bonobos are polymorphic and chimpanzees are fixed for the derived state; (3) both bonobos and chimpanzees are polymorphic; (4) chimpanzees are fixed for the derived state and bonobos are fixed for the ancestral state; and (5) bonobos are fixed for the derived state and chimpanzees are fixed for the ancestral state.**
(DOC)

Table S3 **Location of the selected regions in the human genome.**
(DOC)

## References

[1] Hinds DA, Stuve LL, Nilsen GB, Halperin E, Eskin E (2005). Whole-genome patterns of common DNA variation in three human populations.. Science.

[2] Becquet C, Patterson N, Stone AC, Przeworski M, Reich D (2007). Genetic structure of chimpanzee populations.. PLoS Genet.

[3] Caswell JL, Mallick S, Richter DJ, Neubauer J, Schirmer C (2008). Analysis of chimpanzee history based on genome sequence alignments.. PLoS Genet.

[4] Fischer A, Wiebe V, Pääbo S, Przeworski M (2004). Evidence for a complex demographic history of chimpanzees.. Mol Biol Evol.

[5] Yu N, Jensen-Seaman MI, Chemnick L, Kidd JR, Deinard AS (2003). Low nucleotide diversity in chimpanzees and bonobos.. Genetics.

[6] Fischer A, Pollack J, Thalmann O, Nickel B, Pääbo S (2006). Demographic history and genetic differentiation in apes.. Curr Biol.

[7] Gonder MK, Locatelli S, Ghobrial L, Mitchell MW, Kujawski JT (2011). Evidence from Cameroon reveals differences in the genetic structure and histories of chimpanzee populations.. Proc Natl Acad Sci U S A.

[8] Kaessmann H, Wiebe V, Pääbo S (1999). Extensive nuclear DNA sequence diversity among chimpanzees.. Science.

[9] Braga J (1995). Skeletal variation and measure of divergence among chimpanzees. Contribution of the study of discrete traits.. Académie des sciences.

[10] Shea BT, Leigh SR, Groves CP, Kimbel WH, Martin LB (1993). Multivariate craniometric variation in chimpanzees: implications for species identification;.

[11] Uchida A (1996). What we don't know about great ape variation.. Trends in Ecology and Evolution.

[12] Kano T (1992). The Last Ape: Pygmy Chimpanzee Behavior and Ecology..

[13] Wrangham R, Pilbeam D, Galdikas BMF, Briggs N, Sheeran LK, Shapiro GL, Goodall J (2001). African Apes as time machines.. All Apes Great and Small Vol 1: Chimpanzees, Bonobos and Gorillas.

[14] Zihlman A, McGrew WC, Marchant LF, Nishida T (1996). Reconstruction reconsidered chimpanzee models and human evolution.. Great Ape Societies.

[15] Eriksson J, Hohmann G, Boesch C, Vigilant L (2004). Rivers influence the population genetic structure of bonobos (*Pan paniscus*).. Mol Ecol.

[16] Stone AC, Griffiths RC, Zegura SL, Hammer MF (2002). High levels of Y-chromosome nucleotide diversity in the genus Pan.. Proc Natl Acad Sci U S A.

[17] Becquet C, Przeworski M (2007). A new approach to estimate parameters of speciation models with application to apes.. Genome Res.

[18] Jensen-Seaman MI, Deinard AS, Kidd KK (2001). Modern African ape populations as genetic and demographic models of the last common ancestor of humans, chimpanzees, and gorillas.. J Hered.

[19] Won YJ, Hey J (2005). Divergence population genetics of chimpanzees.. Mol Biol Evol.

[20] Deinard AS, Kidd K (2000). Identifying conservation units within captive chimpanzee populations.. Am J Phys Anthropol.

[21] Voight BF, Adams AM, Frisse LA, Qian Y, Hudson RR (2005). Interrogating multiple aspects of variation in a full resequencing data set to infer human population size changes.. Proc Natl Acad Sci U S A.

[22] Wegmann D, Excoffier L (2010). Bayesian Inference of the Demographic History of Chimpanzees.

[23] Gonder MK, Oates JF, Disotell TR, Forstner MR, Morales JC (1997). A new west African chimpanzee subspecies?. Nature.

[24] Gagneux P, Wills C, Gerloff U, Tautz D, Morin PA (1999). Mitochondrial sequences show diverse evolutionary histories of African hominoids.. Proc Natl Acad Sci U S A.

[25] Bjork A, Liu W, Wertheim JO, Hahn BH, Worobey M (2010). Evolutionary history of chimpanzees inferred from complete mitochondrial genomes.. Mol Biol Evol.

[26] Hudson RR (1992). Gene trees, species trees and the segregation of ancestral alleles.. Genetics.

[27] Pamilo P, Nei M (1988). Relationships between gene trees and species trees.. Molecular Biology and Evolution.

[28] Takahata N (1993). Allelic genealogy and human evolution.. Mol Biol Evol.

[29] Wu CI (1991). Inferences of species phylogeny in relation to segregation of ancient polymorphisms.. Genetics.

[30] Geraldes A, Basset P, Gibson B, Smith KL, Harr B (2008). Inferring the history of speciation in house mice from autosomal, X-linked, Y-linked and mitochondrial genes.. Molecular Ecology.

[31] Xu X, Walters C, Antolin MF, Alexander ML, Lutz S (2009). Phylogeny and biogeography of the eastern Asian-North American disjunct wild-rice genus (*Zizania* L., Poaceae).. Molecular Phylogenetics and Evolution.

[32] McGuire JA, Linkem CW, Koo MS, Hutchinson DW, Lappin AK (2007). Mitochondrial introgression and incomplete lineage sorting through space and time: phylogenetics of crotaphytid lizards.. Evolution.

[33] Heckman KL, Mariani CL, Rosoloarison R, Yoder AD (2007). Multiple nuclear loci reveal patterns of incomplete lineage sorting and complex species history within western mouse lemurs (*Microcebus*).. Molecular Phylogenetics and Evolution.

[34] Hobolth A, Christensen OF, Mailund T, Schierup MH (2007). Genomic relationships and speciation times of human, chimpanzee, and gorilla inferred from a coalescent hidden Markov model.. PLoS Genet.

[35] Patterson N, Richter DJ, Gnerre S, Lander ES, Reich D (2006). Genetic evidence for complex speciation of humans and chimpanzees.. Nature.

[36] Napier JR, Napier PH (1967). A handbook of living primates.

[37] Meyer M, Stenzel U, Hofreiter M (2008). Parallel tagged sequencing on the 454 platform.. Nat Protoc.

[38] Green RE, Krause J, Ptak SE, Briggs AW, Ronan MT (2006). Analysis of one million base pairs of Neanderthal DNA.. Nature.

[39] Brockman W, Alvarez P, Young S, Garber M, Giannoukos G (2008). Quality scores and SNP detection in sequencing-by-synthesis systems.. Genome Res.

[40] Edgar RC (2004). MUSCLE: a multiple sequence alignment method with reduced time and space complexity.. BMC Bioinformatics.

[41] Edgar RC (2004). MUSCLE: multiple sequence alignment with high accuracy and high throughput.. Nucleic Acids Res.

[42] Librado P, Rozas J (2009). DnaSP v5: a software for comprehensive analysis of DNA polymorphism data.. Bioinformatics.

[43] Tajima F (1989). Statistical method for testing the neutral mutation hypothesis by DNA polymorphism.. Genetics.

[44] Kimura M (1983). The neutral theory of evolution..

[45] Hudson RR (2002). Generating samples under a Wright-Fisher neutral model of genetic variation.. Bioinformatics.

[46] Kong A, Gudbjartsson DF, Sainz J, Jonsdottir GM, Gudjonsson SA (2002). A high-resolution recombination map of the human genome.. Nat Genet.

[47] Thornton K (2003). Libsequence: a C++ class library for evolutionary genetic analysis.. Bioinformatics.

[48] Ptak SE, Hinds DA, Koehler K, Nickel B, Patil N (2005). Fine-scale recombination patterns differ between chimpanzees and humans.. Nat Genet.

[49] Winckler W, Myers SR, Richter DJ, Onofrio RC, McDonald GJ (2005). Comparison of fine-scale recombination rates in humans and chimpanzees.. Science.

[50] Pritchard JK, Stephens M, Donnelly P (2000). Inference of population structure using multilocus genotype data.. Genetics.

[51] Patterson N, Price AL, Reich D (2006). Population structure and eigenanalysis.. PLoS Genet.

[52] Ronquist F, Huelsenbeck JP (2003). MrBayes 3: Bayesian phylogenetic inference under mixed models.. Bioinformatics.

[53] Minin V, Abdo Z, Joyce P, Sullivan J (2003). Performance-based selection of likelihood models for phylogeny estimation.. Syst Biol.

[54] Swofford DL (2002). PAUP*.Phylogenetic Analysis Using Parsimony (*and Other Methods). Version 4..

[55] Hasegawa M, Kishino H, Yano T (1985). Dating of the human-ape splitting by a molecular clock of mitochondrial DNA.. J Mol Evol.

[56] Stamatakis A (2006). RAxML-VI-HPC: maximum likelihood-based phylogenetic analyses with thousands of taxa and mixed models.. Bioinformatics.

