# Supplementary figures and images for: Bonobos Fall within the Genomic Variation of Chimpanzees
Source: PLoS One. 2011 Jun 29;6(6):e21605. doi: 10.1371/journal.pone.0021605 (PMC3126833; doi:10.1371/journal.pone.0021605)

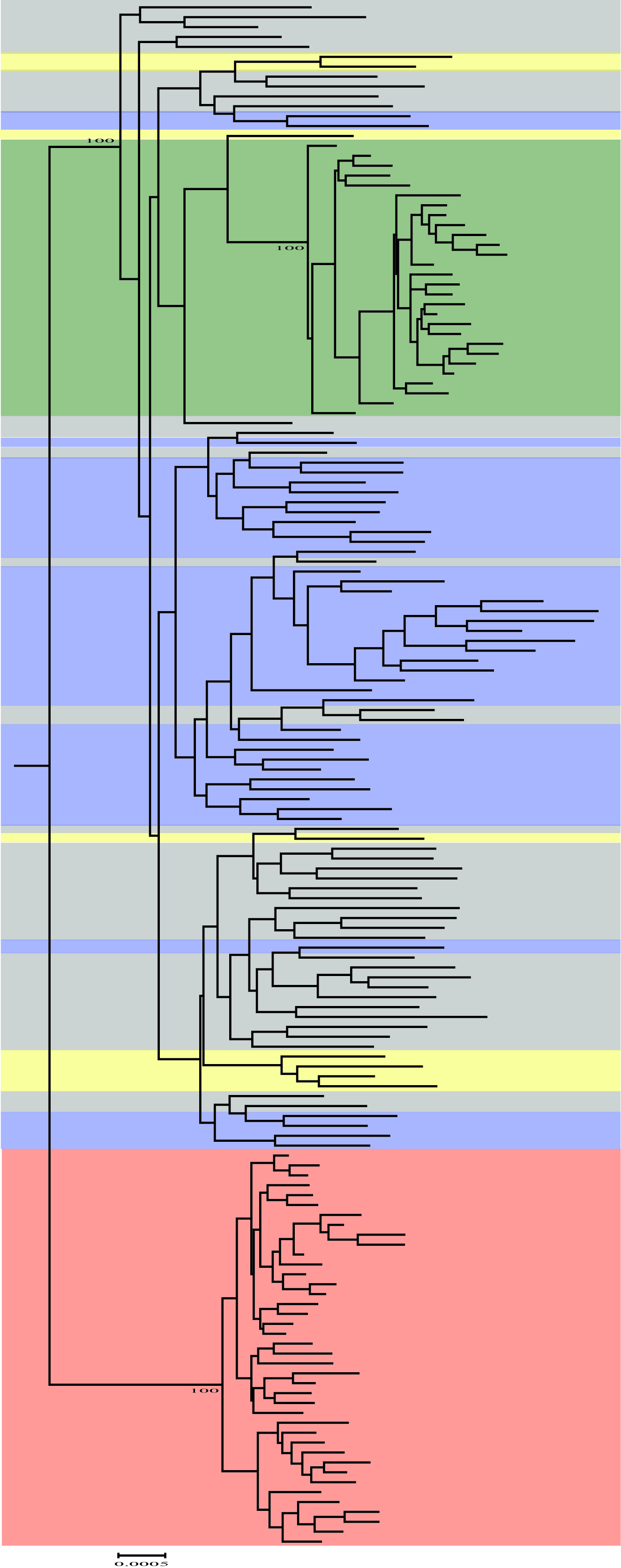

Supplement: Figure S1 — Maximum Likelihood consensus tree based on the concatenated sequences of all 15 regions, with bootstrap values for 2000 replicates. (TIF) [file pone.0021605.s001.tif]

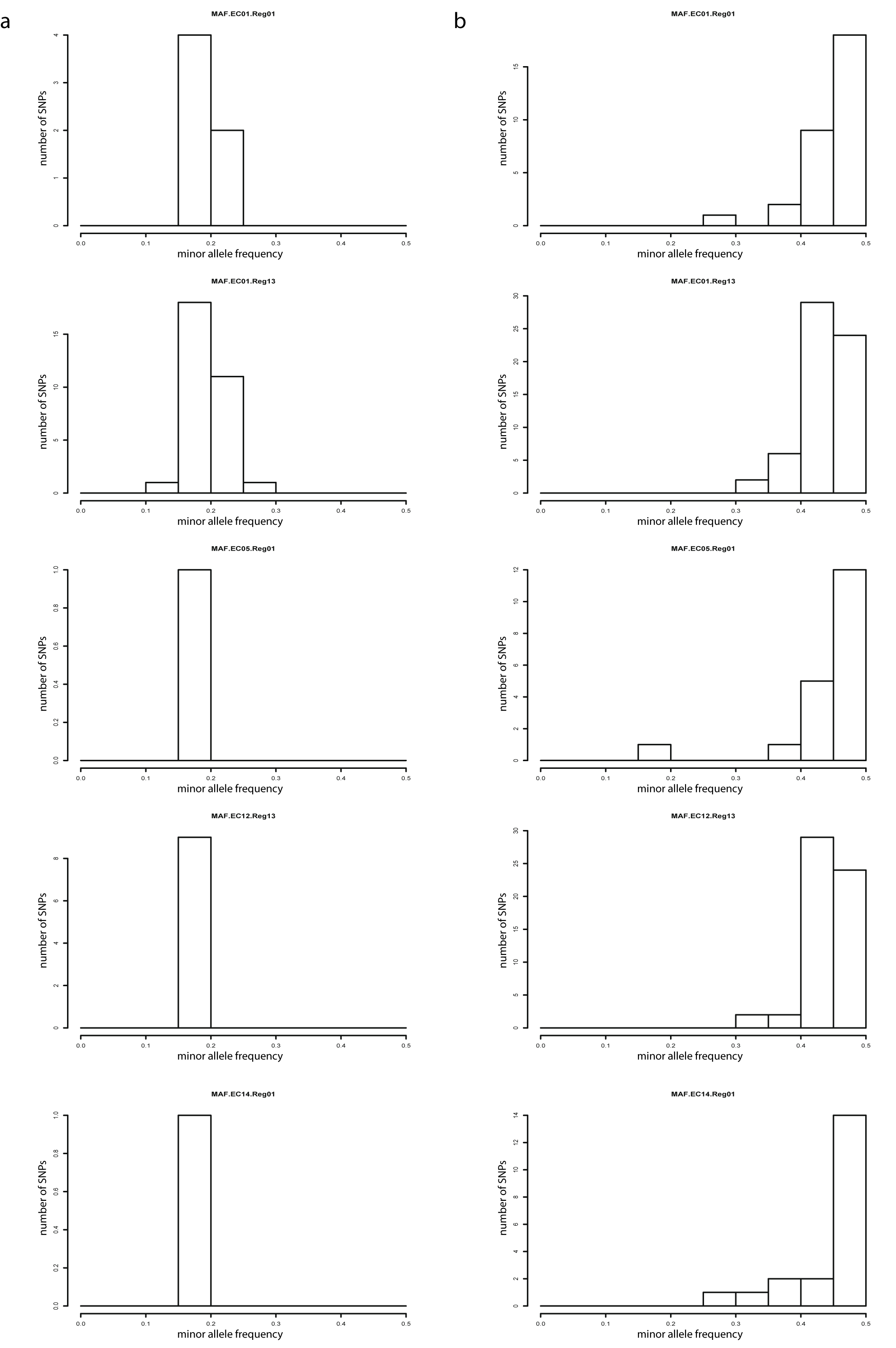

Supplement: Figure S2 — Minor allele frequency distribution for eastern chimpanzees before (a) and after (b) reamplification with a new set of primers. (TIF) [file pone.0021605.s002.tif]
