# Supplementary material for: Bonobos Fall within the Genomic Variation of Chimpanzees
Source: PLoS One. 2011 Jun 29;6(6):e21605. doi: 10.1371/journal.pone.0021605 (PMC3126833; doi:10.1371/journal.pone.0021605)
Supplement: Table S1 — Simulated values of various summary statistics under the standard neutral model matched for S and the number of chromosomes. Also listed is the observed value for each summary statistic. (DOC) [file pone.0021605.s003.doc]

| Species | | Average π | Average Tajima’s D | Variance Tajima’s D | Average Fu and Li’s D^*^ | C |
| --- | --- | --- | --- | --- | --- | --- |
| Bonobo | | | | | | |
|  | Observed | 8.9 | -0.371 | 0.402 | 0.010 | 8.0 |
|  | Median simulated | 9.5 | -0.061 | 0.580 | -0.044 | 6.8 |
|  | p-value | 0.534 | 0.117 | 0.361 | 0.804 | 0.792 |
| Central chimpanzee | | | | | | |
|  | Observed | 22.8 | -0.902 | 0.210 | -1.043 | 59.5 |
|  | Median simulated | 29.7 | -0.045 | 0.379 | -0.035 | 6.8 |
|  | p-value | 0.007 | 0.000 | 0.180 | 0.000 | 0.000 |
| Eastern chimpanzee | | | | | | |
|  | Observed | 19.3 | -0.021 | 0.372 | 0.418 | 10.1 |
|  | Median simulated | 19.3 | -0.052 | 0.455 | -0.043 | 6.9 |
|  | p-value | 0.987 | 0.865 | 0.617 | 0.012 | 0.506 |
| Western chimpanzee | | | | | | |
|  | Observed | 7.5 | -0.148 | 0.610 | 0.105 | 2.5 |
|  | Median simulated | 7.5 | -0.063 | 0.628 | -0.047 | 6.9 |
|  | p-value | 0.970 | 0.673 | 0.931 | 0.463 | 0.128 |
