# Supplementary material for: Bonobos Fall within the Genomic Variation of Chimpanzees
Source: PLoS One. 2011 Jun 29;6(6):e21605. doi: 10.1371/journal.pone.0021605 (PMC3126833; doi:10.1371/journal.pone.0021605)
Supplement: Table S2 — For each region, the number of sites for which (1) chimpanzees are polymorphic and bonobos are fixed for the derived state; (2) bonobos are polymorphic and chimpanzees are fixed for the derived state; (3) both bonobos and chimpanzees are polymorphic; (4) chimpanzees are fixed for the derived state and bonobos are fixed for the ancestral state; and (5) bonobos are fixed for the derived state and chimpanzees are fixed for the ancestral state. (DOC) [file pone.0021605.s004.doc]

| Region | Different classes of sites | | | | |
| --- | --- | --- | --- | --- | --- |
|  | (1) C poly; B all derived | (2) B poly; C all derived | (3) B poly;  C poly | (4) C all derived; B all ancestral | (5) B all derived; C all ancestral |
| (a) | 5 | 0 | 0 | 1 | 5 |
| (b) | 2 | 0 | 1 | 12 | 22 |
| (c) | 5 | 0 | 1 | 0 | 7 |
| (d) | 2 | 0 | 5 | 3 | 6 |
| (e) | 1 | 0 | 1 | 6 | 15 |
| (f) | 3 | 2 | 4 | 0 | 7 |
| (g) | 16 | 0 | 1 | 2 | 13 |
| (h) | 1 | 1 | 3 | 0 | 1 |
| (i) | 4 | 0 | 7 | 0 | 0 |
| (j) | 4 | 0 | 0 | 1 | 8 |
| (k) | 1 | 0 | 0 | 0 | 3 |
| (l) | 6 | 2 | 4 | 0 | 8 |
| (m) | 19 | 0 | 3 | 2 | 7 |
| (n) | 12 | 0 | 2 | 1 | 2 |
| (o) | 4 | 0 | 1 | 0 | 5 |
| Totals | 85 | 5 | 33 | 28 | 109 |
