# Supplementary material for: Bonobos Fall within the Genomic Variation of Chimpanzees
Source: PLoS One. 2011 Jun 29;6(6):e21605. doi: 10.1371/journal.pone.0021605 (PMC3126833; doi:10.1371/journal.pone.0021605)
Supplement: Table S3 — Location of the selected regions in the human genome. (DOC) [file pone.0021605.s005.doc]

| Region | Chromosome | Strand | Start position | End position | Source |
| --- | --- | --- | --- | --- | --- |
| 1 | 12 | + | 46053611 | 46064649 | Voight et al. |
| 2 | 5 | + | 10028764 | 10038991 | Voight et al. |
| 3 | 20 | - | 7611192 | 7622810 | Voight et al. |
| 4 | 5 | - | 128225051 | 128235529 | Voight et al. |
| 5 | 19 | + | 35965170 | 35975708 | Voight et al. |
| 6 | 11 | - | 29506568 | 29517364 | Voight et al. |
| 7 | 5 | - | 62675411 | 62685809 | Voight et al. |
| 8 | 14 | - | 61844214 | 61854882 | Voight et al. |
| 9 | 9 | + | 12050291 | 12060889 | Voight et al. |
| 10 | 18 | - | 10107615 | 10118303 | Voight et al. |
| 11 | 21 | + | 23098338 | 23108836 | Voight et al. |
| 12 | 1 | + | 187167005 | 187177995 | This publication |
| 13 | 2 | + | 5555366 | 5566365 | This publication |
| 14 | 6 | + | 106239404 | 106250404 | This publication |
| 15 | 7 | + | 144943717 | 144954216 | This publication |
